# Successful Application of Tocilizumab in a Patient With Neoadjuvant Immunochemotherapy‐Induced Cytokine Release Syndrome

**DOI:** 10.1002/cnr2.2145

**Published:** 2024-07-25

**Authors:** Soichiro Minami, Yosuke Kawashima, Yasuhiko Munakata, Masahiro Matsuno, Shuichiro Hara, Yusuke Yamazaki, Tsuyoshi Doman, Shin Saito, Tetsuo Odaka, Takahiro Ogasawara, Hisashi Shimizu, Jun Sugisaka, Tomoiki Aiba, Yukihiro Toi, Shinsuke Yamanda, Yuichiro Kimura, Shunichi Sugawara

**Affiliations:** ^1^ Department of Pulmonary Medicine Sendai Kousei Hospital Sendai Miyagi Japan; ^2^ Munakata Yasuhiko Clinic Sendai Miyagi Japan; ^3^ Department of Thoracic Surgery Sendai Kousei Hospital Sendai Miyagi Japan

**Keywords:** adenocarcinoma, cytokine release syndrome, neoadjuvant therapy, nivolumab, tocilizumab

## Abstract

**Background:**

The expansion of preoperative immunochemotherapy has led to an increase in the number of patients with lung cancer receiving immune checkpoint inhibitors (ICIs). Therefore, oncologists should manage a variety of immune‐related adverse events (irAEs). One of the rare, life‐threatening, and recently proposed irAEs is cytokine release syndrome (CRS). Although the standard treatment of irAE is systemic administration of steroids, it has been suggested that tocilizumab may be an effective treatment option for CRS.

**Case:**

This case describes a 69‐year‐old man with stage IIIA lung adenocarcinoma who received chemotherapy and nivolumab, which is an ICI, as neoadjuvant immunochemotherapy. After the first administration, the patient developed severe skin rash, fever, and arthralgia. We suspected irAEs and administered systemic steroids. However, fever and arthralgia did not improve, although the skin rash disappeared. These were also significant challenges for surgery. Noting the elevated levels of inflammatory cytokines, we consulted a rheumatologist. Finally, we decided to terminate neoadjuvant therapy after one cycle and administer tocilizumab. Tocilizumab dramatically improved the patient's symptoms and allowed him to undergo radical surgery. Pathological findings revealed that the patient achieved a major pathological response.

**Conclusion:**

This indicates the potential effectiveness of early tocilizumab administration for ICI‐induced CRS, even in mild cases.

## 1 Introduction

Immune checkpoint inhibitors (ICIs) have been proven effective against various cancers, including lung cancer.

Furthermore, the CheckMate 816 trial showed that the neoadjuvant nivolumab in combination with chemotherapy improved clinical outcomes compared to chemotherapy alone in patients with resectable non‐small cell lung cancer [1].

However, ICIs can activate the immune system and cause immune‐related adverse events (irAEs) in multiple organs, some of which can be life‐threatening [2].

Cytokine release syndrome (CRS), although a rare irAE, can be lethal.

Recently, a Japanese clinical trial (JCOG2007) cautioned against ICI‐induced CRS. The JCOG 2007 trial was a clinical trial to determine the superiority of chemotherapy plus nivolumab and ipilimumab over chemotherapy plus pembrolizumab in advanced or recurrent non‐small cell lung cancer (negative or unknown driver gene mutation). The enrollment began in April 2021 but was terminated in March 2023 due to several deaths related to unanticipated adverse events in the combined use of nivolumab plus ipilimumab. Among treatment‐related deaths, three were diagnosed with CRS [3].

CRS is difficult to diagnose because of its rare occurrence and clinically nonspecific symptoms and signs [4, 5, 6].

In chimeric antigen receptor (CAR) T‐cell therapy‐induced CRS cases, tocilizumab has been widely used for the treatment of any severity in recent years; however, data on the appropriate timing of its administration are scarce [7, 8]. On the other hand, there are a few cases that tocilizumab plus corticosteroid treatment was effective for severe CRS induced by ICIs [9, 10].

Herein, we report a case of neoadjuvant immunochemotherapy‐induced steroid‐resistant CRS that responded to tocilizumab treatment in addition to corticosteroid monotherapy. Pathological findings revealed that the patient achieved a major pathological response (MPR), defined as having less than 10% of viable tumor cells remaining in the primary tumor, following radical surgery.

## 2 Case Presentation

A 69‐year‐old man was referred to our hospital with a lesion in the lower left lung lobe and swollen ipsilateral hilar and subtracheal lymph nodes, as observed in a positron emission tomography/computed tomography (CT) scan. He had been a smoker for 50 years and had no major medical history.

Bronchoscopy was performed on the lung lesion, which revealed lung adenocarcinoma, strongly positive for programmed death‐ligand 1 (tumor proportion score ≥50%) and negative for driver mutations.

The clinical diagnosis was cT2bN2M0, stage IIIA lung adenocarcinoma.

After consultation with a thoracic surgeon, he was placed on neoadjuvant immunochemotherapy and received cisplatin (75 mg/m^2^), pemetrexed (500 mg/m^2^), and nivolumab (360 mg/kg body weight).

However, just 21 days after the first cycle of immunochemotherapy, the patient developed grade 3 skin rash, grade 1 fever, and grade 1 arthralgia as per the Common Terminology Criteria for Adverse Events criteria.

**FIGURE 1:**
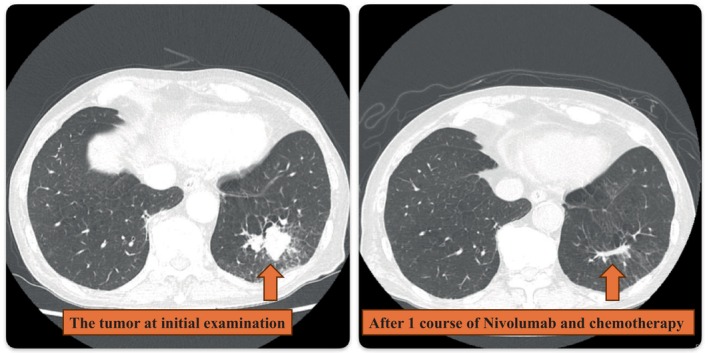
Only one course of nivolumab and chemotherapy resulted in significant shrinkage of the tumor.

We suspected that the cause was noninfectious because a CT scan revealed no signs of infection, and the results of blood and urine culture tests were both negative. As the CT scan showed shrinkage of the primary lung lesion and lymph nodes (Figure 1), we decided to terminate neoadjuvant therapy after one cycle, and radical surgery was planned when the skin rash was controllable.

We considered irAEs and consulted a dermatologist regarding skin rash. In line with the dermatologist's recommendation, we started prednisolone treatment at a dosage of 60 mg (1 mg/kg body weight) along with the most potent steroid ointment.

As the skin rash improved promptly, the dose was tapered off by 20%–30% per few days and a maintenance dose of 7.5 mg was started on day 30 after the administration of steroids.

Although the skin rash had resolved at that point, the patient still had slight fever, arthralgia, and grade 2 anemia (Hb 8.7 g/dL), which posed obstacles to the surgery.

We revisited the patient's pathophysiology again. After gastrointestinal bleeding was ruled out by upper endoscopy and colonoscopy, we emphasized the presence of elevated inflammatory cytokines in the blood test, including a C‐reactive protein (CRP) level of 7.4 mg/dL, a ferritin level of 708 ng/mL, and an interleukin‐6 (IL‐6) level of 61.2 pg/mL. As a result, we sought consultation with a rheumatologist.

After consultation with the rheumatologist, the patient was diagnosed with ICI‐induced CRS because the infection was negative, prolonged fever and arthralgia were observed, anemia appeared, and blood tests showed elevated inflammatory cytokines.

Although the patient did not have hypotension or hypoxemia and had mild CRS (Grade 1 according to the American Society for Transplantation and Cellular Therapy [ASTCT] Consensus Grading [11]), he needed improvement of his general conditions to undergo surgery.

**FIGURE 2:**
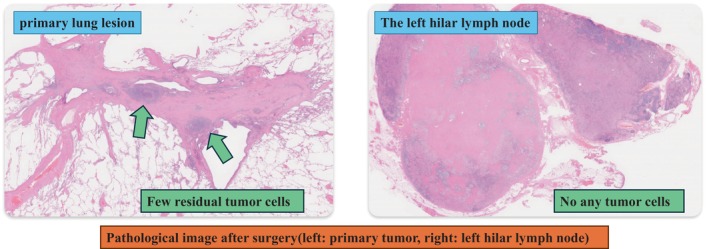
After the surgery, the residual tumor cells represented less than 5% of the primary lung lesion and were not detected in the lymph nodes.

**FIGURE 3:**
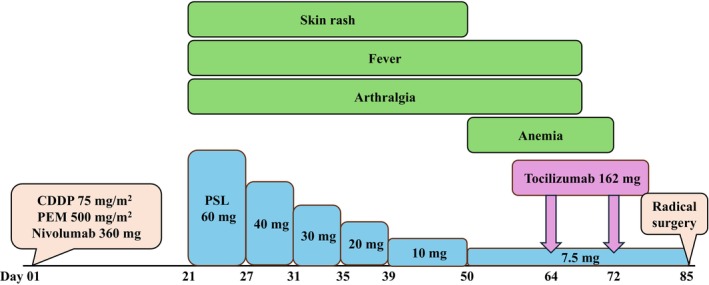
A brief diagram of the patient. Twenty‐one days after the first cycle of immunochemotherapy, the patient developed grade 3 skin rash, grade 1 fever, and grade 1 arthralgia. Prednisolone treatment at a dosage of 60 mg (1 mg/kg body weight) was initiated. Although the skin rash had resolved, the patient still had slight fever, arthralgia, and anemia. After consultation with the rheumatologist, the patient was diagnosed with ICI‐induced CRS and treated with a subcutaneous injection of tocilizumab on days 64 and 72 after the first cycle of immunochemotherapy. With tocilizumab treatment, the patient's symptoms substantially improved. On day 85 after the first cycle of immunochemotherapy, he underwent radical surgery. CDDP, cisplatin; PEM, pemetrexed; PSL, prednisolone.

For this reason, tocilizumab administration was recommended. The patient was treated with a subcutaneous injection of tocilizumab (162 mg, biweekly) on days 64 and 72 after the first cycle of immunochemotherapy, and the symptoms substantially improved. Moreover, the results of blood test showed significant improvement in anemia (from 8.7 to 10.0 g/dL) and CRP (from 7.4 to 0.03 mg/dL). On day 85 after the first cycle of immunochemotherapy, he underwent radical surgery, and pathological findings confirmed that residual tumor cell proportions were less than 5% in the primary lung lesion, and no malignant cells were detected in the lymph nodes (Figure 2). The patient exhibited an MPR. A brief diagram of the clinical course is shown in Figure 3.

## 3 Discussion

In this case, the poor general condition due to CRS affected operative tolerance despite the mild severity. Following the administration of tocilizumab, there was a remarkable improvement in the patient's overall condition, thus enabling the patient to undergo radical surgery.

CAR‐T‐cell therapy is an immunotherapy with proven antitumor effects [12, 13].

While ICI strongly blocks inhibitory signals that suppress T‐cell activity and reactivate tumor immunity by T cells, CAR‐T therapy genetically engineers a patient's T cells to express chimeric antigen receptors that can directly recognize and target specific tumor antigens [14]. Both ICI and CAR‐T therapies are similar in that they reactivate tumor immunity by T cells [15].

In patients experiencing CRS after treatment with CAR‐T therapy, CRS is widely treated with tocilizumab, even in mild cases [7]. Although there is no established consensus on the indication and timing of tocilizumab treatment in ICI‐induced CRS, tocilizumab may be considered in mild cases, as in this case.

This study had two limitations. First, in the CheckMate 816 trial, 93.8% of patients received three cycles of nivolumab plus platinum‐doublet chemotherapy [1]. In this case, the patient received only one cycle because of adverse effects. The long‐term prognosis of the patient is unknown, although an MPR was observed after surgery. Second, the clinical course of patients with CRS remains unknown, which is a concern because CRS is a life‐threatening irAE. Following surgery, oncologists should carefully observe patients while cooperating with rheumatologists.

## 4 Conclusion

This case highlights the use of tocilizumab in addition to steroids to manage symptoms associated with ICI‐induced CRS. Although the severity of CRS was mild, he needed improvement of his general conditions to undergo surgery. Consequently, the patient's general condition improved with tocilizumab, and he achieved MPR after undergoing surgery. This implies the potential effectiveness of early administration of tocilizumab for ICI‐induced CRS even in mild cases.

## Author Contributions

**Soichiro Minami:** writing – original draft. **Yosuke Kawashima:** writing – original draft. **Yasuhiko Munakata:** writing – review and editing. **Masahiro Matsuno:** writing – review and editing. **Shuichiro Hara:** writing – review and editing. **Yusuke Yamazaki:** writing – review and editing. **Tsuyoshi Doman:** writing – review and editing. **Shin Saito:** writing – review and editing. **Tetsuo Odaka:** writing – review and editing. **Takahiro Ogasawara:** writing – review and editing. **Hisashi Shimizu:** writing – review and editing. **Jun Sugisaka:** writing – review and editing. **Tomoiki Aiba:** writing – review and editing. **Yukihiro Toi:** writing – review and editing. **Shinsuke Yamanda:** writing – review and editing. **Yuichiro Kimura:** writing – review and editing. **Shunichi Sugawara:** writing – review and editing.

## Ethics Statement

The authors have nothing to report.

## Consent

Written informed consent was obtained from the patient for publishing this case report and accompanying images.

## Conflicts of Interest

Yosuke Kawashima received personal fees from Taiho Pharmaceutical, Eli Lilly, Life Technologies Japan Ltd, Chugai Pharma, AstraZeneca, and Kyowa Kirin. Yukihiro Toi received personal fees from AstraZeneca, Chugai Pharma, Pfizer, Taiho Pharmaceutical, Kyowa Kirin, Bristol‐Myers Squibb, Ono Pharmaceutical, and MSD K.K. Shinsuke Yamanda received personal fees from AstraZeneca, Novartis, Sanofi, GSK, and Nippon Boehringer Ingelheim. Shunichi Sugawara received personal fees from AstraZeneca, Chugai Pharma, Pfizer, Taiho Pharmaceutical, Eli Lilly, Novartis, Kyowa Kirin, Bristol‐Myers Squibb, Ono Pharmaceutical, MSD K.K, and Nippon Boehringer Ingelheim. All other authors declare no conflict of interest.

## Data Availability

The data that support the findings of this study are available from the corresponding author upon reasonable request.

## References

[1] P. M. Forde , J. Spicer , S. Lu , et al., “Neoadjuvant Nivolumab Plus Chemotherapy in Resectable Lung Cancer,” New England Journal of Medicine 386 (2022): 1973–1985. 35403841 10.1056/NEJMoa2202170 PMC9844511

[2] D. Y. Wang , J. E. Salem , J. V. Cohen , et al., “Fatal Toxic Effects Associated With Immune Checkpoint Inhibitors: A Systematic Review and Meta‐Analysis,” JAMA Oncology 4, no. 12 (2018): 1721–1728. 30242316 10.1001/jamaoncol.2018.3923 PMC6440712

[3] Y. Shiraishi , T. Tokito , R. Toyozawa , et al., “Five Cases of Cytokine Release Syndrome in Patients Receiving Cytotoxic Chemotherapy Together With Nivolumab Plus Ipilimumab: A Case Report,” Journal of Thoracic Oncology 19, no. 2 (2024): 337–343. 37943237 10.1016/j.jtho.2023.10.010

[4] A. Ceschi , R. Noseda , K. Palin , and K. Verhamme , “Immune Checkpoint Inhibitor‐Related Cytokine Release Syndrome: Analysis of WHO Global Pharmacovigilance Database,” Frontiers in Pharmacology 11 (2020): 557. 32425791 10.3389/fphar.2020.00557 PMC7212758

[5] P. Gödel , A. Shimabukuro‐Vornhagen , and M. von Bergwelt‐Baildon , “Understanding Cytokine Release Syndrome,” Intensive Care Medicine 44 (2018): 371–373. 28956093 10.1007/s00134-017-4943-5

[6] T. Tsutsui , K. Hata , M. Kawaguchi , H. Kobayashi , Y. Kakizaki , and Y. Miyashita , “Cytokine Release Syndrome Complicated With Severe Rashes Induced by Nivolumab Plus Ipilimumab Therapy in a Patient With Non‐Small Cell Lung Cancer: A Case Report,” Thoracic Cancer 14 (2023): 2310–2313. 37381088 10.1111/1759-7714.15015 PMC10423655

[7] Y. Zhang , F. Zhou , Z. Wu , et al., “Timing of Tocilizumab Administration Under the Guidance of IL‐6 in CAR‐T Therapy for R/R Acute Lymphoblastic Leukemia,” Frontiers in Immunology 13 (2022): 914959. 35799791 10.3389/fimmu.2022.914959 PMC9253384

[8] D. W. Lee , R. Gardner , D. L. Porter , et al., “Current Concepts in the Diagnosis and Management of Cytokine Release Syndrome,” Blood 124 (2014): 188–195. 24876563 10.1182/blood-2014-05-552729 PMC4093680

[9] S. H. Tay , M. M. X. Toh , Y. L. Thian , et al., “Cytokine Release Syndrome in Cancer Patients Receiving Immune Checkpoint Inhibitors: A Case Series of 25 Patients and Review of the Literature,” Frontiers in Immunology 13 (2022): 807050. 35154124 10.3389/fimmu.2022.807050 PMC8831742

[10] S. J. Rotz , D. Leino , S. Szabo , J. L. Mangino , B. K. Turpin , and J. G. Pressey , “Severe Cytokine Release Syndrome in a Patient Receiving PD‐1‐Directed Therapy,” Pediatric Blood & Cancer 64, no. 12 (2017): e26642, 10.1002/pbc.26642. 28544595

[11] D. W. Lee , B. D. Santomasso , F. L. Locke , et al., “ASTCT Consensus Grading for Cytokine Release Syndrome and Neurologic Toxicity Associated With Immune Effector Cells,” Biology of Blood and Marrow Transplantation 25, no. 4 (2019): 625–638. 30592986 10.1016/j.bbmt.2018.12.758 PMC12180426

[12] R. C. Sterner and R. M. Sterner , “CAR‐T Cell Therapy: Current Limitations and Potential Strategies,” Blood Cancer Journal 11, no. 4 (2021): 69. 33824268 10.1038/s41408-021-00459-7 PMC8024391

[13] S. L. Maude , N. Frey , P. A. Shaw , et al., “Chimeric Antigen Receptor T Cells for Sustained Remissions in Leukemia,” New England Journal of Medicine 371 (2014): 1507–1517. 25317870 10.1056/NEJMoa1407222 PMC4267531

[14] B. Long , E. Brém , and A. Koyfman , “Oncologic Emergencies: Immune‐Based Cancer Therapies and Complications,” Western Journal of Emergency Medicine 21, no. 3 (2020): 566–580. 32421502 10.5811/westjem.2020.1.45898 PMC7234690

[15] C. H. June , R. S. O'Connor , O. U. Kawalekar , S. Ghassemi , and M. C. Milone , “CAR T Cell Immunotherapy for Human Cancer,” Science 359, no. 6382 (2018): 1361–1365. 29567707 10.1126/science.aar6711

